# Peers in Primary Care: How Peer Specialists Support Aging Veterans

**DOI:** 10.1093/geroni/igaa057.2154

**Published:** 2020-12-16

**Authors:** Amanda Peeples, Anjana Muralidharan, Lorrianne Kuykendall, Richard Goldberg, Matthew Chinman

**Affiliations:** 1 US Department of Veterans Affairs, Baltimore, Maryland, United States; 2 VA Capitol Healthcare Network, Baltimore, Maryland, United States; 3 Veterans Affairs Capitol Healthcare Network, Baltimore, Maryland, United States; 4 US Department of Veterans Affairs, Pittsburgh, Pennsylvania, United States

## Abstract

Most of the more than 1,100 peer specialists (“peers”) in the Veterans Health Administration (VHA) work in mental healthcare settings. These peers provide a variety of services to Veterans such as facilitating groups, teaching recovery and coping skills, connecting Veterans with VHA and community services, and helping Veterans navigate VHA care. In 2014 the White House issued an Executive Action mandating the reassignment of peers from mental health to primary care settings in 25 VHA locations nationwide. This paper presents qualitative findings from a mixed-methods study evaluating this implementation of peers in VHA primary care. We found that peers assisted aging Veterans in primary care through activities such as providing health coaching, facilitating health education groups, connecting Veterans with services, and providing general peer support. Findings are drawn from semi-structured interviews with 27 peers, 25 supervisors, and 10 Veterans who received services from the peers in primary care.

